# Diversity of mobile genetic elements in carbapenem-resistant Enterobacterales isolated from the intensive care units of a tertiary care hospital in Northeast India

**DOI:** 10.3389/fmicb.2025.1543427

**Published:** 2025-05-22

**Authors:** Shravani Mitra, Sharmi Naha, Joy Chakraborty, Subhadeep De, Harpreet Kaur, Tapan Majumdar, Sulagna Basu

**Affiliations:** ^1^Division of Bacteriology, ICMR-National Institute for Research in Bacterial Infections (Formerly ICMR-NICED), Kolkata, West Bengal, India; ^2^Department of Microbiology, Agartala Government Medical College & G B Pant Hospital, Agartala, Tripura, India; ^3^Division of Communicable Diseases, Indian Council of Medical Research (ICMR), New Delhi, India

**Keywords:** plasmids, insertion sequence element, transposons, *bla*_NDM-1,5,7_, *bla*_OXA-181,232_, *bla*_KPC-2_-harboring *Escherichia coli*, core genome phylogeny

## Abstract

**Introduction:**

Mobile genetic elements (MGEs) play a crucial role in the spread of carbapenem resistance. A study was undertaken to characterize MGEs and evaluate their contribution to the spread of carbapenem resistance in *Escherichia coli* and *Klebsiella pneumoniae* collected from three intensive care units (ICUs) of a tertiary care hospital in Tripura.

**Methods:**

Isolates were subjected to susceptibility testing, genotypic detection of carbapenemases and their transmissibility, whole-genome sequencing (WGS), and phylogenomic analysis.

**Results:**

*E. coli* and *K. pneumoniae* were the dominant Enterobacterales, exhibiting resistance to the majority of antibiotics. WGS of carbapenemase-producing *E. coli* (*n* = 15/48,31%) and *K. pneumoniae* (*n* = 13/26,50%) revealed the presence of *bla*_NDM-1,5,7_ (*n* = 21), *bla*_KPC-2_ (*n* = 1), and *bla*_OXA-181,232_ (*n* = 8). Isolates were diverse and belonged to different sequence types, including epidemic clones (*K. pneumoniae*-ST16/101/147/231; *E. coli*-ST167/410/648). This study has noted the allelic shift of *bla*_NDM-1_ to *bla*_NDM-5_ similar to global reports. *bla*_NDM-1,5,7_-bearing plasmids were conjugative but those carrying *bla*_KPC-2_ and *bla*_OXA-181,232_ were non-conjugative. *bla*_NDM-1,5,7_ were present in diverse replicons: IncF-types (predominant), IncHI1B, IncX3, and IncX4, etc., while *bla*_OXA-181,232_ were present in ColKP3, corroborating with global studies. *bla*_NDM-1,5_ was associated with intact/truncated IS*Aba125* in Tn*125*, *bla*_NDM-7_ with IS*3000*, *bla*_KPC-2_ with IS*Kpn6*, and IS*Kpn7* in Tn*4401*b, and *bla*_OXA-181,232_ with ∆IS*Ecp1* in Tn*2013*, depicting an ancestral genetic context noted globally. Study isolates were related to other Indian isolates, primarily from blood.

**Discussion:**

The association with different MGEs noted in the study is similar to those in other parts of India and the globe, signifying that the genetic determinants are part of the global gene pool. These associations can facilitate the spread of carbapenem resistance, leading to outbreaks and treatment failures.

## 1 Introduction

Bacteria share genes through vertical (parent to offspring) and horizontal (bacterium to bacterium) gene transfer. Genes transferred horizontally are typically not essential for basic cellular functions; instead, they are often accessory genes such as antibiotic resistance genes (ARGs). Spread of ARGs is often mediated by certain discrete DNA sequences known as mobile genetic elements (MGEs) (Partridge et al., 2018). Components of MGEs, such as plasmids and integrative conjugative elements (ICEs), enable intercellular mobility of DNA, while insertion sequence (IS) elements, transposons (Tn), and integrons (In) facilitate intracellular DNA mobility (Partridge et al., 2018; Khedkar et al., 2022). Dissemination of MGEs is facilitated by horizontal gene transfer (HGT) mechanisms (conjugation, transformation, transduction, and vesiduction), which ultimately leads to bacterial evolution (da Silva et al., 2021). Occurrence of ARGs (*bla*_CTX-M_, *bla*_NDM_, *bla*_KPC_, *bla*_OXA-48-like_, etc.) within the MGEs leads to the establishment of multidrug resistance (MDR) within the bacterial community, rendering available antibiotics ineffective (Liu et al., 2024).

Carbapenem, the last resort drug to treat severe MDR infections, have become ineffective due to the emergence of various carbapenemase genes such as class-A serine carbapenemase: *Klebsiella pneumoniae* carbapenemase (KPC; *bla*_KPC_); class-B metallo-*β*-lactamase (MBL): New Delhi metallo-β-lactamase (NDM; *bla*_NDM_) and class-D serine carbapenemase: oxacillinases (OXA)-48 and its variants (*bla*_OXA-48-like_) (Cui et al., 2019). KPC, NDM, and OXA-48 were first reported in *K. pneumoniae* and have subsequently been reported in *Escherichia coli* as well as various genera of Gram-negative bacteria (Pitout et al., 2019; Wu et al., 2019; Ding et al., 2023). These three carbapenemases differ from each other in many attributes. KPC and OXA-48 contain serine at the active site, while NDM contains zinc ions. The hydrolysis profile of these enzymes for the majority of β-lactam antibiotics (benzylpenicillin, ampicillin, piperacillin, cephalothin, temocillin, first-generation cephalosporins, and carbapenems) is similar, except for the fact that KPC and OXA-48 cannot hydrolyze cefoxitin and ceftazidime, but NDM can hydrolyze all. Although KPC and OXA-48 are considered carbapenemases, their hydrolysis potential for carbapenems is not similar to NDM; that is, both KPC and OXA-48 can hydrolyze meropenem sparingly, and OXA-48 exhibits the highest catalytic activity toward imipenem. These carbapenemases have been reported across the globe from different sources, namely, human, environment (soil, water, etc.), companion animals, and so on (Taggar et al., 2020). However, the propensity of occurrence differs for each carbapenemase (Al-Zahrani and Alsiri, 2018). NDM is the widely reported carbapenemase (Wu et al., 2019), while KPC is endemic to North and South America, China, and parts of Europe (Ding et al., 2023), and OXA-48 is prevalently reported from Middle East countries. Carbapenemases are borne of diverse plasmid replicons, which aid in their successful spread across the globe (Pitout et al., 2019). They are often associated with various MGEs, such as IS*Aba125*, IS*Ecp1*, Tn*3*, Tn*125*, Tn*4401*, and Tn*2013* (Partridge et al., 2018).

The presence of these carbapenemases on different mobilizable plasmids has led to the spread of these genes across the globe (Lee et al., 2016; Wu et al., 2019; Ding et al., 2023). *bla*_NDM_ is associated with a broad range of plasmid types (IncA/C, IncF, IncR, IncN, IncH, IncL/M, IncX, IncB/O/K/Z, IncY, IncT, and ColE10) (Lee et al., 2016; Wu et al., 2019) compared to *bla*_KPC_ (IncF, IncFII, IncFIIK, IncI2, IncX, IncA/C, IncR, and ColE1) (Wyres and Holt, 2018; MacKinnon et al., 2020; Gorrie et al., 2022) and *bla*_OXA-48-like_ (IncL/M, IncF, IncA/C, IncHI, IncI, IncX, IncN, IncX3, IncT, and ColE-type) (Pitout et al., 2019). In addition, these genes are linked with various IS elements, such as IS*Aba125*, IS*6*, and IS*CR27* (*bla*_NDM_); IS*Kpn6*, IS*Kpn7*, IS*Kpn8* (*bla*_KPC_); and IS*1999* and its variants, IS*Ecp1* and IS*1R* (*bla*_OXA-48-like_) (Pitout et al., 2019; Acman et al., 2022), which have facilitated their spread within the bacterial community. Also, the association of these carbapenemases has been found with various transposons, such as *bla*_NDM_ with Tn*3*, Tn*125*, and Tn*3000*; *bla*_KPC_ with Tn*4401*b and its variants; and *bla*_OXA-48-like_ with Tn*1999*, Tn*6237*, and Tn*2013* (Pitout et al., 2019; Acman et al., 2022). The contribution of MGEs in the spread of these genes is well exemplified by the global dissemination of *bla*_NDM-1_, *bla*_KPC_, and *bla*_OXA-48-like_ within different members of Enterobacterales, especially *E. coli* and *K. pneumoniae* (Lee et al., 2016; Pitout et al., 2019; Acman et al., 2022).

*E. coli* and *K. pneumoniae* are critical pathogens as per the World Health Organization (WHO) priority pathogen list,^1^ and are known to cause a wide range of community-acquired and healthcare-associated infections among adults as well as neonates (Wyres and Holt, 2018; MacKinnon et al., 2020; Gorrie et al., 2022). They are efficient in acquiring ARGs (Paczosa and Mecsas, 2016), and a recent study has suggested that the *K. pneumoniae* genome is an “open pangenome” with more than 400 acquired AGRs, highlighting increased HGT rate in this species (Navon-Venezia et al., 2017; Martin and Bachman, 2018; Wyres and Holt, 2018; Wang et al., 2023). With the acquisition of carbapenem-resistant genes via different MGEs, *E. coli* and *K. pneumoniae* have become resistant to carbapenems.

In this study, carbapenem-resistant *E. coli* and *K. pneumoniae* isolated from different intensive care units (ICUs) of a tertiary care hospital of Northeast India (Tripura) were analyzed in terms of diversity of bacterial etiology, susceptibility, carbapenemase genes, transmissibility of carbapenemases and MGEs (plasmids, IS elements, transposons) associated with it. Despite the antibiotic exposure typical of ICUs, it remains uncertain which MGEs primarily facilitate the dissemination of resistance genes. The study site is in Northeast India, where incidences of antimicrobial resistance (AMR) have been explored in a minimal way. The Northeast India is heterogeneous in terms of its geographical location, high rainfall, forest coverage, and diverse ethnic groups. This leads to compromised healthcare facilities resulting in morbidity, mortality, and increased AMR.^2^ Since the assessment of MGEs in the spread of carbapenem resistance is crucial in containing AMR spread, this study was conducted.

## 2 Methods

### 2.1 Ethics approval and consent for participation

The study protocol was approved by the Institutional Ethics Committee of the Indian Council of Medical Research (ICMR)-National Institute for Research in Bacterial Infections (formerly, ICMR-NICED) (A-1/2019-IEC.). Patient consent was taken prior to enrolment in the study. Patient information was anonymized and de-identified prior to analysis. All experiments were performed in accordance with relevant guidelines and regulations.

### 2.2 Isolation, identification, and antibiotic susceptibility testing of study isolates

Clinical specimens (blood, urine, and cerebrospinal fluid) were collected from patients admitted to the anesthesia intensive care unit (AICU), pediatric intensive care unit (PICU), and neonatal intensive care unit (NICU) of Agartala Government Medical College, Agartala, Tripura, India, from October 2019 to January 2021. Isolates were identified using VITEK2 COMPACT system (BioMérieux, Marcy-l’Étoile, France). Antibiotic susceptibilities of the study Enterobacterales were determined using the VITEK^®^2 AST-N281 card (BioMérieux, Marcy-l’Étoile, France). Minimum inhibitory concentration (MIC) of colistin was separately determined by broth microdilution using a commercial colistin MIC test kit (MIKROLATEST, Erba Lachema s.r.o., Czech Republic). Additionally, the MIC of meropenem (carbapenem) was assessed using E-strip (BioMérieux, Marcy-l’Étoile, France). The results were interpreted according to Clinical and Laboratory Standards Institute guidelines (CLSI, 2024). Isolates showing intermediate susceptibility values to any antimicrobials were considered non-susceptible to that antimicrobial.

### 2.3 Detection of carbapenemase producers and determining their genetic relatedness

A multiplex polymerase chain reaction (PCR) (Grönthal et al., 2018) was carried out to detect carbapenemases genes: *bla*_KPC_, *bla*_IMP_, *bla*_VIM_, *bla*_NDM_, and *bla*_OXA-48_ within the studied isolates, followed by sequencing the genes using primers mentioned in earlier studies (Yigit et al., 2001; Espinal et al., 2011). Genetic relatedness of the carbapenemase-producing Enterobacterales was studied by pulsed-field gel electrophoresis (PFGE) using a CHEF-DRIII apparatus (Bio-Rad Laboratories, Inc., Hercules, CA, United States) as described previously (Tenover et al., 1995). Processing of the PFGE images and the preparation of the dendrogram were performed by FP Quest software v4.5 (Bio-Rad Laboratories, Inc., Hercules, CA, United States) using the unweighted pair group method with arithmetic mean (UPGMA) and Dice coefficient. Isolates having more than 95% similarity were considered identical. *Salmonella Braenderup* H9812 isolate was used as a molecular weight marker. PFGE for one isolate (AGA0088) could not be performed, but other characterization of this isolate was performed.

### 2.4 Conjugation assay and study of the transferred plasmid types

Transmissibility of carbapenemase genes was assessed by conjugation using wild-type isolates as donors and the azide-resistant *E. coli* J53 isolate as the recipient. Transconjugants (TCs) were selected on Luria Agar plates (BD Diagnostics, Franklin Lakes, NJ, USA) supplemented with cefoxitin (10 mg/L), ertapenem (0.25 mg/L), and sodium azide (100 mg/L) (Sigma–Aldrich, St. Louis, Missouri, United States). TCs were screened for the presence of carbapenemase genes along with other resistance genes. Replicon types of the wild-types and TCs were detected using the PCR-based replicon typing (PBRT) kit.^3^

### 2.5 Whole-genome sequencing and genotypic characterization of carbapenemase producers in terms of sequence types, serotype, antimicrobial resistance, and virulence genes, and plasmid types

Whole genome sequencing of all carbapenemase-producing Enterobacterales was performed. DNA library was prepared with the genomic DNA using Nextera XT Kit (Illumina, San Diego, CA, USA), followed by sequencing in the Illumina Novaseq 6000 instrument (Illumina, San Diego, CA, USA). Contig assembly was carried out by Unicycler (Wick et al., 2017) and Shovill.^4^ Annotation was performed by RAST.^5^ Galaxy Project Europe (see text footnote 4) was used to analyze the genomic data. Antimicrobial resistance genes were identified using Staramar (see text footnote 4) and CARD.^6^ Plasmid types were determined by Inctyper of pathogenwatch^7^ and PlasmidFinder.^8^ Multilocus Sequence Typing (MLST) was determined by Pathogenwatch (see text footnote 7), and for *E. coli*, the Warwick scheme was followed. Serotyping of *K. pneumoniae* was determined by Kleborate (see text footnote 7), and for *E. coli*, via SeroTypeFinder.^9^

### 2.6 Study of genetic environment, IS elements, and transposons associated with carbapenemase genes

The genetic environment of the carbapenemase genes was determined by SnapGene viewer (v8) and National Center for Biotechnology Information (NCBI) Blast using WGS data. To determine the upstream region of carbapenemase genes, primer walking was performed wherever required, due to the constraint of contig size as a result of short-read sequencing. The presence of mobile genetic elements within the study genomes was analyzed by the mobile element finder (MGE) server.^10^

### 2.7 Core genome phylogenetic analysis

Two types of phylogenetic trees were prepared for (i) comparison of study isolates among themselves for both the genera under study and (ii) comparison of study isolates (both genera) with isolates from different parts of India and neighboring countries close to the study site. *E. coli* and *K. pneumoniae* isolated from different parts of India and neighboring countries, such as Bangladesh and Myanmar (depending on availability of genomes), possessing *bla*_NDM-1,5,7_, *bla*_KPC-2_, and *bla*_OXA-181,232_ submitted to the NCBI database were downloaded (last accessed: 15 February 2025) and used for the comparative analysis. For this analysis, other isolates (non-study isolates) were selected on the basis of their isolation sources, that is, blood and urine, as the study isolates were of blood and urine origin. Along with this, host diseases (bacteremia, sepsis, neonatal sepsis, urinary tract infections), availability of assembly files, complete AMR genotypes, and sequence types specific to the study isolates were also considered. Phylogenetic trees were built using Roary (v3.13.0) (https://usegalaxy.eu/?tool_id=roary), and the resulting Newick files were visualized and annotated in iTOL (v7) (https://itol.embl.de/).

## 3 Results

### 3.1 Diversity of bacterial isolates collected from different ICUs

**Figure 1:**
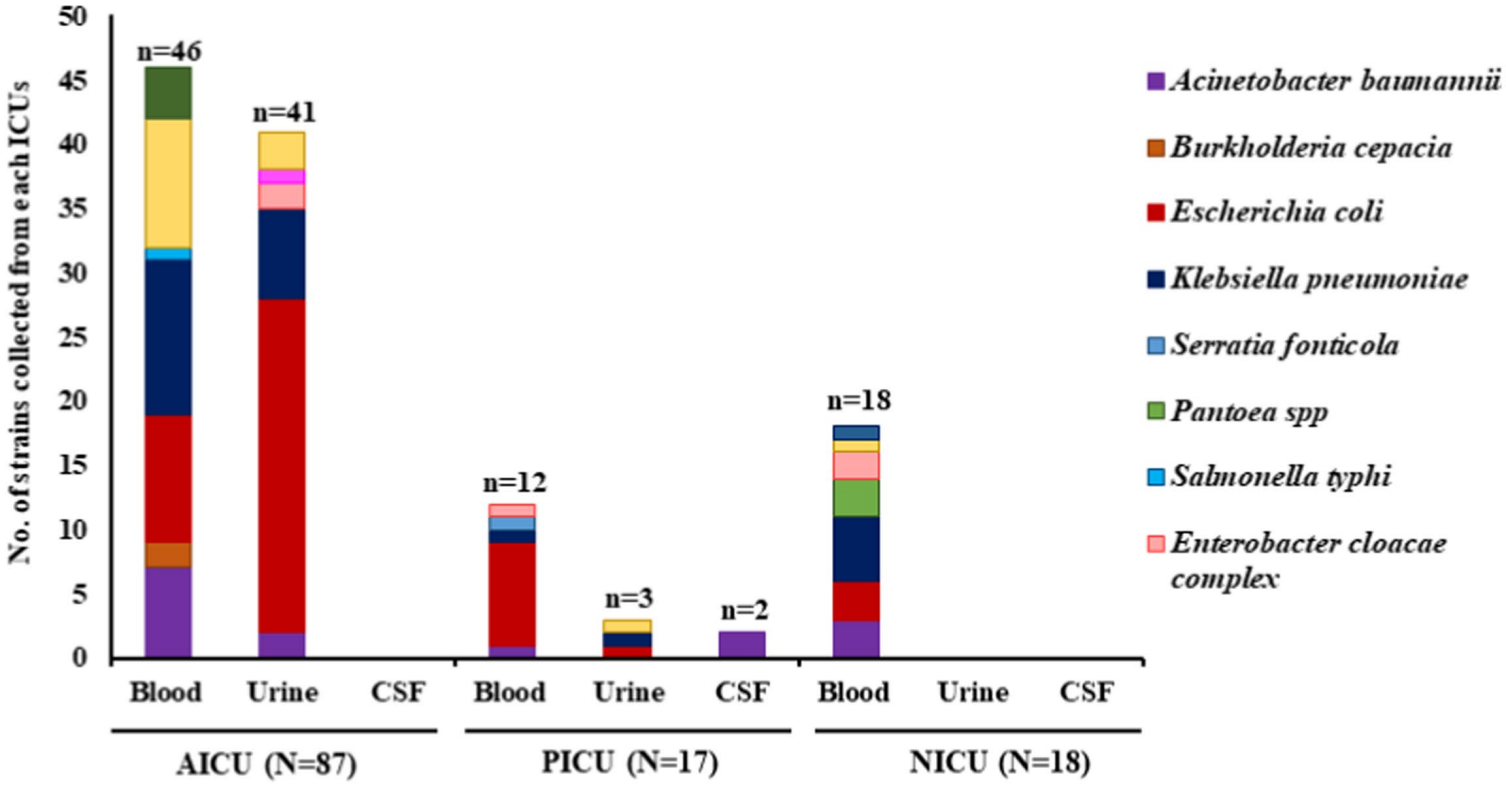
Schematic diagram of Gram-negative organisms recovered from different intensive care units along with their source of isolation. AICU, Anesthesia intensive care unit; PICU, pediatric intensive care unit; NICU, neonatal intensive care unit; CSF, cerebrospinal fluid; N = total number of isolates in each ICU; n = number of isolates in each specimen.

Of 286 culture-positive isolates collected from the AICU, PICU, and NICU of Agartala Government Medical College, Agartala, Tripura, India, 122 isolates were identified as Gram-negative bacteria. Of them, *E. coli* (*n* = 48, 39%) and *K. pneumoniae* (*n* = 26, 21%) were the dominant ones. Other Gram-negative bacteria identified were *Acinetobacter baumannii* (*n* = 15), *Pseudomonas aeruginosa* (*n* = 16), *Enterobacter cloacae* complex (*n* = 5), *Proteus mirabilis* (*n* = 4), *Pantoea* sp. (*n* = 3), *Burkholderia cepacia* (*n* = 2), *Salmonella typhi* (*n* = 1), *Shigella sonnei* (*n* = 1), *Serratia fronticola* (*n* = 1) (Figure 1). The collection of isolates was higher in AICU (*n* = 87) compared to that in NICU (*n* = 18) and PICU (*n* = 17). Isolates were collected mainly from the bloodstream (*n* = 76), urinary infections (*n* = 44), and a few from cerebrospinal fluid (*n* = 2) (Table 1). *E. coli* was isolated from both urine (*n* = 27) and blood (*n* = 21), while *K. pneumoniae* was mainly from blood (*n* = 18) and a few from urine (*n* = 8). Four *K. pneumoniae* isolated from blood were lost upon subculturing. Hence, the major Enterobacterales, *E. coli* and *K. pneumoniae* (*n* = 70), were further analyzed.

**Table 1:** Detailed breakup of carbapenemases found in study isolates, units, and source of isolation.

Organism	AICU (*n* = 24)	PICU (*n* = 3)	NICU (*n* = 1)
*E. coli* (*n* = 15)	NDM-5 = 10; U = 10	NDM-5 = 2; B = 2	NF
	NDM-7 = 2; U = 2		
	KPC-2 = 1; U = 1		
*Klebsiella pneumoniae* (*n* = 13)	NDM-1 = 1; B = 1	NDM-1 = 1; B = 1	NDM-1 = 1; B = 1
	NDM-5 = 2; B = 1; U = 1		
	OXA-181 = 2, B = 2		
	OXA-232 = 4; B = 2; U = 2		
	OXA-181 + NDM-5 = 2; B = 2		

AICU, Anesthesia intensive care unit; PICU, pediatric intensive care unit; NICU, neonatal intensive care unit; B, blood; U, urine; n, number of isolates; NF, not found.

### 3.2 Antimicrobial susceptibility and genotypic characterization of study isolates collected from ICUs

*E. coli* and *K. pneumoniae* (*n* = 70) exhibited varying level of non-susceptibility toward different groups of antimicrobials (% resistant *K. pneumoniae* vs. % resistant *E. coli*) such as piperacillin-tazobactam: 73% vs. 56%; doripenem, imipenem, meropenem: 68% vs. 40%; amikacin: 55% vs. 17%; ciprofloxacin: 77% vs. 90%; levofloxacin: 77% vs. 94%; trimethoprim-sulphamethoxazole: 41% vs. 63%; and colistin: 14% vs. 2% (Supplementary Table S1). *K. pneumoniae* isolates exhibited higher non-susceptibility toward carbapenems compared to *E. coli*. Twenty-eight Enterobacterales, that is, *E. coli* (*n* = 15) and *K. pneumoniae* (*n* = 13), exhibited non-susceptibility toward the carbapenem group of drugs (doripenem, imipenem, and meropenem). They were assessed for the presence of carbapenemase genes by conventional PCR and validated by whole-genome sequencing (WGS).

**Figure 2:**
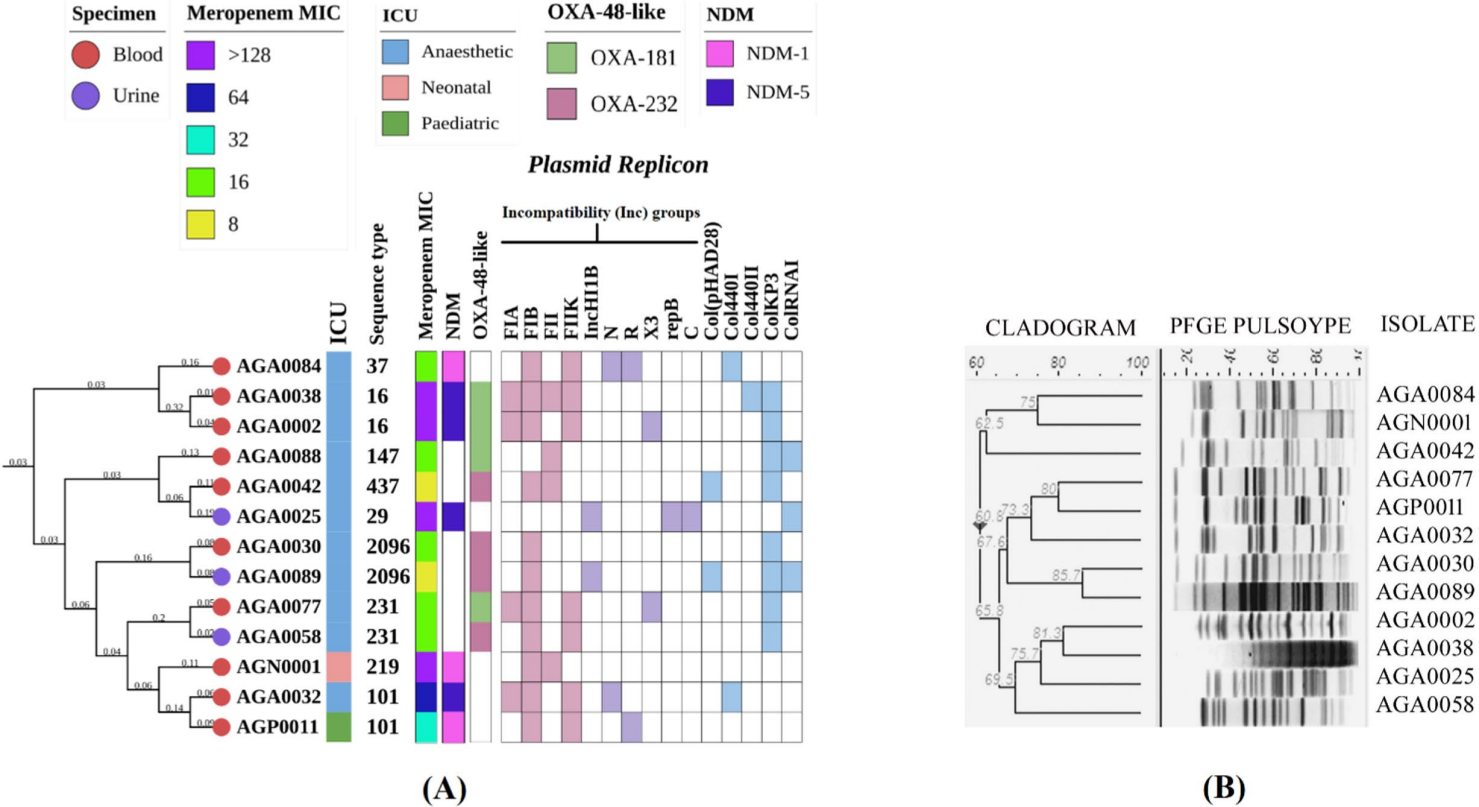
Molecular typing, core genome phylogeny, and genomic characterization of carbapenem-resistant *Klebsiella pneumoniae* isolated from different ICUs. **(A)** Core genome phylogeny of study isolates among themselves along with their source of isolation, specimen type, sequence types, meropenem MIC, carbapenemase genes, and replicons, **(B)** Analysis of pulsed-field gel electrophoresis using XbaI digestion pattern based on Dice’s similarity coefficient and UPGMA (the position tolerance and optimization was set at 1.5 and 1.5%, respectively). MIC, Minimum inhibitory concentration; ICU, Intensive care unit. PFGE for AGA0088 could not be performed, but WGS for this isolate was done, and hence it was included in the core phylogeny.

**Figure 3:**
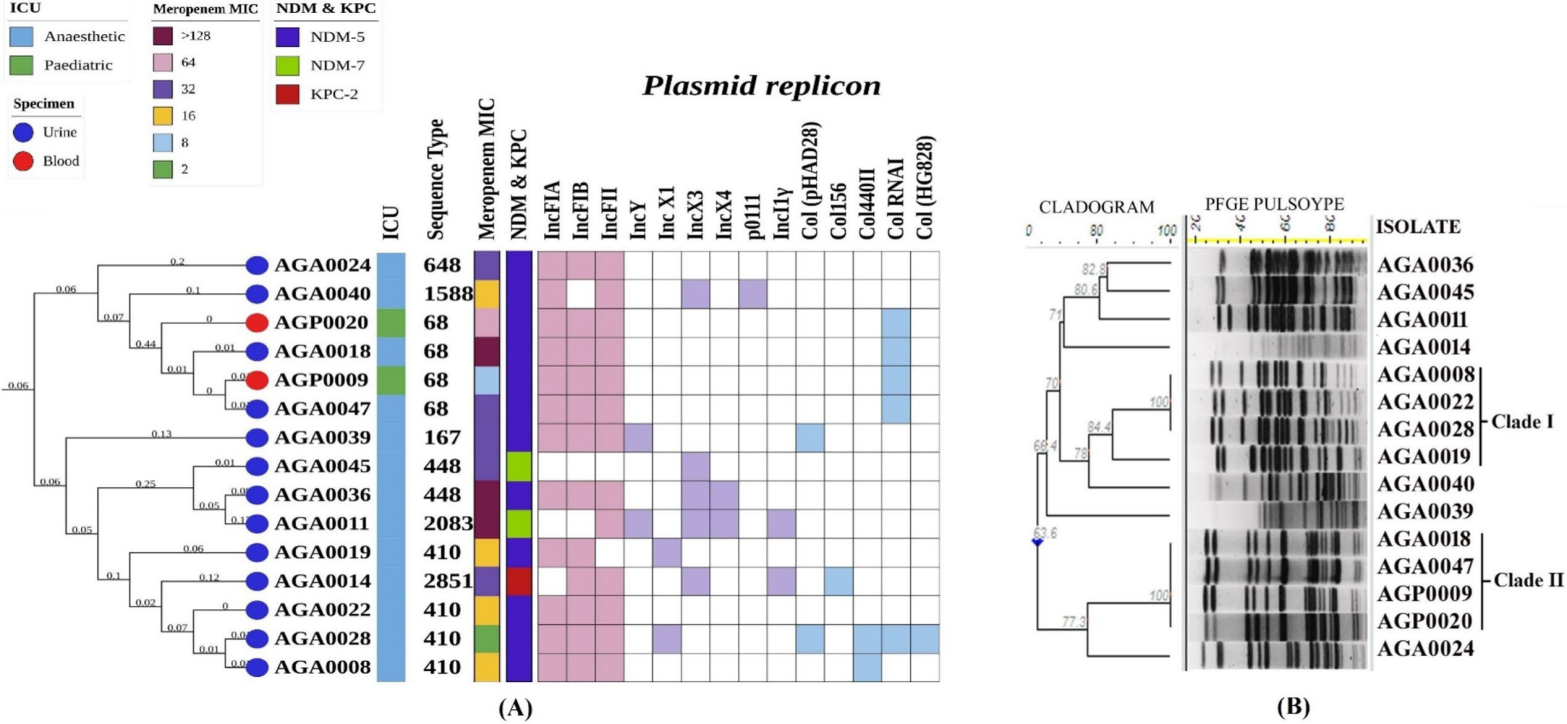
Molecular typing, core genome phylogeny, and genomic characterization of carbapenem-resistant *E. coli* isolated from different ICUs. **(A)** Core genome phylogeny of study isolates among themselves along with their source of isolation, specimen type, sequence types, meropenem MIC, carbapenemase genes, and replicons, **(B)** Analysis of pulsed-field gel electrophoresis using XbaI digestion pattern based on Dice’s similarity coefficient and UPGMA (the position tolerance and optimization was set at 1.5 and 1.5% respectively). Abbreviations: MIC: Minimum inhibitory concentration; ICU: Intensive care unit.

Three types of carbapenem-resistant genes, namely, *bla*_KPC-2_ (class-A serine carbapenemase), *bla*_NDM_ (class-B metallo-*β*-lactamase), and *bla*_OXA-48-like_ (class-D serine carbapenemase), were noted among the ICUs. Three variants of *bla*_NDM_ were found, namely, *bla*_NDM-1_ (*n* = 3/28), *bla*_NDM-5_ (*n* = 16/28), and *bla*_NDM-7_ (*n* = 2/28). *bla*_NDM-5_ was the dominant variant and was mainly identified in *E. coli* (*n* = 12) collected from urine (*n* = 10) and in a few *K. pneumoniae* (*n* = 4) in blood (*n* = 3), urine (*n* = 1) (Table 1). *bla*_NDM-7_ was found only in uropathogenic *E. coli* at AICU (Table 2). Two variants of *bla*_OXA-48-like_: *bla*_OXA-181_ (*n* = 4) and *bla*_OXA-232_ (*n* = 4) were identified in *K. pneumoniae* only, from blood. Two *K. pneumoniae* isolates (AGA0002 & AGA0038) from AICU exhibited the presence of dual carbapenemases, *bla*_NDM-5_ with *bla*_OXA-181_. One *E. coli* (AGA0014) harbored *bla*_KPC-2_; however, none of the *E. coli* harbored *bla*_OXA-48-like_ carbapenemase (Table 1; Figures 2A, 3A). Isolates were resistant to meropenem, with MICs ranging between 8 and >128 mg/L (Figures 2A, 3A; Tables 2, 3), and isolates harboring dual carbapenemases (*bla*_NDM-5_ and *bla*_OXA-181_) exhibited an increased meropenem MIC (>128 mg/L) (Figure 2A; Table 2).

**Table 2:** Genotypic characterization, transmissibility of carbapenemases, and their association with mobile genetic elements among carbapenemase-producing *Klebsiella pneumoniae* isolates collected from anesthesia, pediatric, and neonatal intensive care units.

Isolates	Source	Sequence type (ST)	K- and O-antigen types	Conjugation results	Meropenem MIC (mg/L)	Resistance determinants present and transferred	Carbapenemases present and transferred	Mobile genetic elements (MGEs)		
								Plasmid replicon types present and associated with carbapenemases as per WGS data	IS element present and associated with carbapenemaseses per WGS data	Transposon (Tn) present and associated with carbapenemases per WGS data
AGA0002	Blood	ST16	K81, O13	Successful	>128	*oqxA, oqxB, aac(3)-IIa, aac(6′)-Ib-cr, aadA2, aph(3″)-Ib, aph(6)-Id, bla*_CTX-M-15_ *, bla*_OXA-1_ *, bla*_SHV-148_ *, bla*_TEM-1B_ *, catB3, dfrA12, dfrA14, mph(A), qacE, qnrS1, rmtB, sul1, sul2.*	*bla*_NDM-5_, *bla*_OXA-181_	IncFIA, IncFIB, IncX3, IncFIIK, **ColKP3** (OXA-181)	IS*Kpn14*, IS*Kox1*, IS*Kpn42*, IS*Ech12*, IS*Sty2*, IS*Kpn21*, IS*102,* IS*Aba125*, **IS*Ecp1*** (OXA-181)	Tn*6196,* Tn*125*
				AGA0002.T1	8	*rmtB*	*bla*_NDM-5_	**IncFIA, IncFIB, IncFIIK, IncX3** (NDM)	**IS*Aba125*** (NDM)	**Tn*125*** (NDM)
AGA0030	Blood	ST2096	K64, O1a	Unsuccessful	16	*oqxA, oqxB, aac(6′)-Ib-cr, bla*_OXA-1_ *, bla*_SHV-100_ *, catB3, dfrA1, dfrA12, fosA6, sul1.*	*bla*_OXA-232_	IncFIB(K), **ColKP3** (OXA-232)	IS*Sen3*, IS*6100*, IS*Kox1*, IS*Kpn1*, IS*26*, **∆IS*Ecp1*** (OXA-232)	**∆Tn*2013*** (OXA-232)
AGA0032	Blood	ST101	K2, O1	Successful	64	*arr-2, oqxA, oqxB, ant(3″)-Ia, aph(3″)-Ib, aph(6)-Id, bla*_CTX-M-15_ *, bla*_SHV-100_ *, bla*_TEM-1B_ *, cmlA1, dfrA14, ere(A), qacE, qnrS1, rmtB, sul1, sul2, tet(A).*	*bla*_NDM-5_	IncFIA(HI1), IncFIB(K), IncN, IncFIIK, Col440I	IS*Kpn19*, IS*5075*, IS*Ec9*, IS*EcI1*, IS*903*, IS*102*, IS*Sty2*, IS*Spu2*, IS*6100,* IS*Aba125*	Tn*5403,* Tn*125*
				AGA0032.T1	32	-	*bla*_NDM-5_	**IncN** (NDM)	**IS*Aba125*** (NDM)	**Tn*125*** (NDM)
AGA0038	Blood	ST16	K81, O13	Successful	>128	*oqxA, oqxB, aadA2, bla*_CTX-M-15_ *, bla*_SHV-148_ *, bla*_TEM-1B_ *, dfrA12, mph(A), qacE, qnrS1, rmtB, sul1.*	*bla*_NDM-5_, *bla*_OXA-181_	IncFIA, IncFIB(K), IncFII, IncFIIK, Col440II, **ColKP3** (OXA-181)	IS*Kpn26*, IS*Kpn19*, IS*6100*, IS*Kpn14*, ISKpn33, IS*5075*, IS*Ecl1*, IS*Kox3*, IS*Aba125,* **IS*Ecp1*** (OXA181)	Tn*125*
				AGA0038.T1	0.5	*bla*_CTX-M-15_	*bla*_NDM-5_	Non-typable	**IS*Aba125*** (NDM)	**Tn*125*** (NDM)
AGA0042	Blood	ST437	K36, O4	Unsuccessful	8	*oqxA, oqxB, bla*_CTX-M-15_ *, bla*_SHV-182_ *, dfrA30, fosA6, qacE.*	*bla*_OXA-232_	IncFIB, IncFII, **ColKP3** (OXA-232), Col(pHAD28)	IS*Kpn38*, IS*VSa5*, IS*Kpn25*, IS*Ec9*, IS*Kox1*, IS*Kpn1*, IS*Kpn18*, IS*26*, **∆IS*Ecp1*** (OXA-232)	**∆Tn*2013*** (OXA-232)
AGA0077	Blood	ST231	K51, O1ab	Unsuccessful	16	*arr-2, oqxA, aac(6′)-Ib-Hangzhou, aac(6′)-Ib-cr, aadA2, bla*_CTX-M-15_ *, bla*_SHV-100_ *, bla*_TEM-1B_ *, catA1, dfrA12, mph(A), qacE, qnrS1, rmtF, sitABCD, sul1.*	*bla*_OXA-181_	IncFIA, IncFIB, IncFIIK, IncX3, **ColKP3** (OXA-181)	IS*Sty2*, IS*Kpn14*, IS*Ec33*, IS*Kpn25*, IS*Kox1*, IS*903*, IS*Kpn38*,ISKpn19, IS*Sen3*, IS*Ec36*, IS*Kox3*, ∆IS*Ecp1*(OXA-181)	**∆Tn*2013*** (OXA-181)
AGA0084	Blood	ST37	K14, O3b	Successful	16	*oqxA, oqxB, bla*_CTX-M-15_ *, bla*_SHV-81_ *, bla*_TEM-1B_ *, dfrA1, fosA6, qacE, qnrS1, sul1, tet(A).*	*bla*_NDM-1_	IncFIB(K), IncFIIK, IncR, IncN, Col440I	IS*Ec9*, IS*Kpn1*, IS*Kpn38*, IS*Ec52*, IS*6100*, ∆IS*Aba125*	∆Tn*125*
				AGA0084.T1	16	*bla*_CTX-M-15_*, bla*_SHV-81_*, bla*_TEM-1B_, *qnrS1*	*bla*_NDM-1_	**IncN** (NDM)	**∆IS*Aba125*** (NDM)	**∆Tn*125*** (NDM)
AGA0088	Blood	ST147	K64, O2a	Unsuccessful	16	*oqxA, oqxB, aac(6′)-Ib-Hangzhou, aac(6′)-Ib-cr, bla*_CTX-M-15_ *, bla*_SHV-67_ *, dfrA12, fosA, qnrB1, rmtF.*	*bla*_OXA-181_	IncFII, ColRNAI**, ColKP3** (OXA-181)	IS*Kpn43*, IS*Sen3*, IS*6100*, IS*Kox1*, IS*Kpn1*, IS*26*, **∆IS*Ecp1*** (OXA-181)	**∆Tn*2013*** (OXA-181)
AGN0001	Blood	ST219	K144, O1ab	Successful	>128	*oqxA, oqxB, aac(3)-IId, aadA2, aph(3″)-Ib, aph(3′)-Ia, aph(3′)-VI, aph(6)-Id, bla*_CTX-M-15_, *bla*_DHA-7_, *bla*_SHV-148,_ *dfrA12, fosA, mph(A), qacE, qnrB1, qnrS1, rmtC, sul1, sul2*	*bla*_NDM-1_	IncFIB, IncFIB(K), IncFII	IS*Aba14*, IS*Kpn19*, IS*Cfr1*, IS*6100*, IS*Kpn25*, IS*Ec9*, IS*Eam1*, IS*Kpn24*, IS*Aba125*	Tn*125*
				AGN0001.T1	32	*bla*_CTX-M-15_, *bla*_DHA-7_,*bla*_SHV-148,_ *qnrB1, oqxA*, *qnrS1, rmtC*	*bla*_NDM-1_	**IncFIB, IncFII (NDM)**	**IS*Aba125*** (NDM)	**Tn*125*** (NDM)
AGP0011	Blood	ST101	Unknown (Best match KL106), O1ab	Successful	32	*arr-3, oqxA, oqxB, aac(6′)-Ib, aac(6′)-Ib-cr, aac(6′)-Ib3, aadA1, aph(3″)-Ib, aph(3′)-VI, aph(6)-Id, bla*_CTX-M-15_ *, bla*_OXA-9_ *, bla*_SFO-1_ *, bla*_SHV-100_ *, bla*_TEM-1B_ *, qnrS1, rmtF, sul2.*	*bla*_NDM-1_	IncFIB, IncFIB(K), IncFIIK, IncR	IS*Kpn19*, IS*Ec9*, IS*26*, IS*Kpn14*, IS*6100*, IS*Ecl1*, IS*Sty2*, IS*Kpn21*, IS*102*, IS*Kpn26*, IS*Ec52*, IS*5*, IS*Kpn1*, IS*Kpn8,* IS*Aba125*	Tn*5403*, Tn*6082,* Tn*125*
				AGP0011.T1	32	*oqxA, aac(6′)-Ib-cr,* *bla*_CTX-M-15_*, bla*_SHV-100_*, bla*_TEM-1B_*, qnrS1*	*bla*_NDM-1_	**IncFIIK, IncR (NDM)**	**IS*Aba125* (NDM)**	**Tn*125* (NDM)**
AGA0025	Urine	ST29	K19, O1/O2v2	Successful	>128	*oqxA, oqxB, aac(6′)-Ib-cr, aac(6′)-Ib3, bla*_CMY-6_ *, bla*_CTX-M-15_ *, bla*_SHV-187_ *, fosA6, qacE, rmtC, sul1.*	*bla*_NDM-5_	IncC, repB, IncHI1B, ColRNAI	IS*Kpn14*, IS*Kox1*, IS*Kpn42*, IS*Ech12*, IS*Sty2*, IS*Kpn21*, IS*102, ∆*IS*Aba125*	***∆*Tn*125***
				AGA0025.T1	8	*aac(6′)-Ib-cr, rmtC*	*bla*_NDM-5_	**IncHI1B (NDM)**	***∆*IS*Aba125* (NDM)**	***∆*Tn*125* (NDM)**
AGA0058	Urine	ST231	K51, O1/O2v2	Unsuccessful	16	*arr-3, oqxA, oqxB, aac(6′)-Ib-Hangzhou, aac(6′)-Ib-cr, bla*_CTX-M-15_, *bla*_SHV-100_, *bla*_TEM-1B_, *catA1, qnrS1, rmtF.*	*bla*_OXA-232_	IncFIB, IncFIIK, **ColKP3** (OXA-232)	IS*6100*, IS*Kpn19*, IS*Ec33*, IS*Kpn25*, IS*Kox1*, IS*Sty2*, IS*Sen4*, IS*903*,IS*Kpn38*, IS*Sen3*, IS*Ec36*, IS*Kpn14,* ***∆*IS*Ecp1*** (OXA-232)	**∆Tn*2013*** (OXA-232)
AGA0089	Urine	ST2096	K64, O13	Unsuccessful	8	*oqxA, oqxB, aac(6′)-Ib-cr, aadA2, bla*_CTX-M-15_ *, bla*_SHV-28_ *, dfrA12, fosA6, qacE, sul1, tet(D).*	*bla*_OXA-232_	IncFIB (pNDM-MAR), IncHI1B (pNDM-MAR), ColRNAI, **ColKP3 (OXA-232),** Col(pHAD28)	IS*682*, IS*Ec28*, IS*Kpn26*, IS*Kpn28*, IS*26*	Tn*6196*

MIC, Minimum inhibitory concentration; IS, Insertion sequence; WGS, genome sequencing; T, Transconjugants. IS elements, replicons, and transposons associated with carbapenemases have been bold faced.

**Table 3:** Genotypic characterization, transmissibility of carbapenemases, and their association with mobile genetic elements among carbapenemase-producing *E. coli* isolates collected from anesthesia, pediatric, and neonatal intensive care units.

Isolates	Source	Sequence type (ST)	Phylogroup	O- and H-antigen serotypes	Conjugation results	Meropenem MIC (mg/L)	Resistance determinants present and transferred	Carbapenemases present and transferred	Mobile genetic elements		
									Plasmid replicon types present and associated with carbapenemases as per WGS data	IS element present and associated with carbapenemasesas per WGS data	Transposon (Tn) present and associated with carbapenemases per WGS data
AGA0008	Urine	ST410	C	O:H9	Unsuccessful	16	*aadA2, bla*_CTX-M-15_ *, dfrA12, mph(A), sul1, tet(A)*	*bla*_NDM-5_	IncFIA, IncFIB, IncFII, Col440II	IS30, IS*Prre1*, IS*911*, IS*621*, IS*609*, MITE*Ec1*, IS*Kpn14*, IS*Kpn8*, IS*621*, MITE*Ype1*, IS*Ecl3*, IS*Ec1*, IS*Ec30*, IS*609*, IS*679*, IS*Sm1*, IS*640*, IS*Ec27*, IS*682*, IS*5*, IS*629*, IS*Ec38*, IS*Kpn42*, IS*6100*, IS*Ec9*, IS*26*, IS*Ec27,* **IS*Aba125* (NDM)**	**Tn*125* (NDM)**, Tn*1999*, Tn*5403*
AGA0011	Urine	ST2083	B1	O1:H6	Successful	>128	*bla*_CMY-42_ *, bla*_SFO-1_ *, bla*_TEM-1B_ *, mph(A), rmtB*	*bla*_NDM-7_	IncFII, IncY, IncX3, IncX4, IncI1γ	MITE*Ec1*, IS*4*, IS*5*, IS*26*, IS*911*, IS*5075*, IS*6100*, IS*Ec1*, IS*Ec31*, IS*Ec33*, IS*Ec38*, IS*Ec45,* IS*Ec81*, IS*Kox3, ∆*IS*Aba125*	Tn*6170*, ∆Tn*125*
					AGA0011.T1	1	*bla*_CMY-42_	*bla*_NDM-7_	**IncX4, IncI1γ (NDM)**	***∆*IS*Aba125* (NDM)**	**∆Tn*125* (NDM)**
AGA0014	Urine	ST2851	C	O180:H18	Unsuccessful	32	*aph(3″)-Ib, aph(6)-Id, bla*_CTX-M-15_ *, bla*_TEM-1B_ *, dfrA14, sul2, tet(A)*	*bla*_KPC-2_	IncFIB, IncFII, Col156, IncX3, IncI1γ	IS*3*, IS*26*, IS*30,* IS*629*, IS*5075*, MITE*Ec1*, IS*Ec9*, IS*Ec38*, IS*Kpn8*	Tn*2*, **Tn*4401*b (KPC-2)**
AGA0018	Urine	ST68	D	O99:H6	Successful	128	*aac(6′)-Ib-cr, aac(6′)-Ib-cr, aadA2, aadA5, bla*_CTX-M-15_ *, bla*_OXA-1_ *, bla*_TEM-1B_ *, catA1, catB4, dfrA12, dfrA17, mph(A), rmtB, sul1, tet(B)*	*bla*_NDM-5_	IncFIA, IncFIB, IncFII, ColRNAI	IS*3*, IS*5*, IS*26*, IS*102*, IS*150*, IS*609*, IS*629*, IS*911*, IS*6100*, IS*Ecl10*, MITE*Ec1*, MITE*Ype1*, IS*Ec1*, IS*Ec5*, IS*Ec9*, IS*Ec13*, IS*Ec18*, IS*Ec27*, IS*Ec30*, IS*Ec31*, IS*Ec38*, IS*Ec46*, IS*Ec47*, IS*Shes15*, IS*Sfl11*, IS*Sham1*, IS*Ech5*, IS*Ersp1*, IS*Sfl10*, IS*Spu2*, IS*Eic2*, IS*Prre1*, IS*Kpn14*, IS*Kpn42*, IS*Kpn47,* IS*Aba125*	Tn*2*, Tn*125,* Tn*801*, Tn*1999*, Tn*6196*
					AGA0018.T1	8	*bla*_CTX-M-15_ *, bla*_TEM-1B_ *, rmtB*	*bla*_NDM-5_	**IncFII (NDM)**	**IS*Aba125* (NDM)**	**Tn*125* (NDM)**
AGA0019	Urine	ST410	C	O8:H9	Unsuccessful	16	*aac(3)-IId, aac(6′)-Ib-cr, aadA2, aadA5, bla*_CMY-54_ *, bla*_CTX-M-15_ *, bla*_OXA-1_ *, bla*_TEM-57_ *, catB4, dfrA12, dfrA17, mph(A), rmtB, sul1, tet(B)*	*bla*_NDM-5_	IncFIA, IncFIB, Inc. X1	IS*4*, IS*30*, IS*100*, IS*6100*, MITE*Ec1,* IS*Aba125*	**Tn*125* (NDM)**
AGA0022	Urine	ST410	C	O:H9	Unsuccessful	16	*aadA2, bla*_CTX-M-15_ *, dfrA12, mph(A), sul1, tet(A)*	*bla*_NDM-5_	IncFIA, IncFIB, IncFII	IS*5*, IS*26*, IS*30*, IS*6100*, IS*Kpn8*, MITE*Ec1*, IS*Ec38,* IS*Aba125*	**Tn*125* (NDM)**, Tn*5403*
AGA0024	Urine	ST648	F	O8:H4	Successful	32	*aac(3)-IIa, aac(6′)-Ib-cr, aadA2, bla*_CMY-42_ *, bla*_CTX-M-15_ *, bla*_OXA-1_ *, bla*_TEM-1B_ *, catB4, dfrA12, erm(B), mph(A), sul1*	*bla*_NDM-5_	IncFIA, IncFIB, IncFII	IS*5*, IS*6100*, IS*Ec31*, IS*Ec45*, MITE*Ec1, ∆*IS*Aba125*	∆Tn*125*
					AGA0024.T1	32	*aac(6′)-Ib-cr, bla*_CMY-42_ *, bla*_CTX-M-15_ *, bla*_OXA-1_ *, bla*_TEM-1B_	*bla*_NDM-5_	**IncFIA, IncFIB, IncFII (NDM)**	***∆*IS*Aba125* (NDM)**	**∆Tn*125* (NDM)**
AGA0028	Urine	ST410	C	O:H9	Successful	16	*aadA2, aph(3″)-Ib, aph(3′)-Ia, aph(6)-Id, bla*_CTX-M-15_ *, dfrA12, mph(A), qnrS1, sul1, sul2, tet(A)*	*bla*_NDM-5_	IncFIA, IncFIB, IncFII, Col (pHAD28), Col440II, Col RNAI, Col (HG828), Inc. X1	IS*5*, IS*26*, IS*30*, IS*102*, IS*5075*, IS*6100*, IS*Ec1*, IS*Ec38*, IS*Kpn8*, IS*Kpn19*, MITE*Ec1,* IS*Aba125*	Tn*125* (NDM), Tn*5403*
					AGA0028.T1	16	*bla*_CTX-M-15_ *, qnrS*	*bla*_NDM-5_	**IncFIA, IncFIB, IncFII (NDM)**	**IS*Aba125* (NDM)**	**Tn*125* (NDM)**
AGA0036	Urine	ST448	B1	O160:H8	Successful	>128	*aac(6′)-Ib-cr, aadA5, bla*_CTX-M-15_ *, bla*_SFO-1_ *, bla*_TEM-1B_ *, dfrA17, mph(A), sul1, tet(B)*	*bla*_NDM-5_	IncFIA, IncFIB, IncFII, IncX3	IS*4*, IS*5*, IS*30*, IS*100* IS*102*, IS*679*, IS911, IS*5075*, IS*6100*, IS*Ec1*, IS*Kox3*, MITE*Ec1,* IS*Aba125*	Tn*125*
					AGA0036.T1	16	-	*bla*_NDM-5_	**IncX3, IncFIA, IncFIB, IncX4 (NDM)**	**IS*Aba125* (NDM)**	**Tn*125* (NDM)**
AGA0039	Urine	ST167	Unknown	O8:H9	Successful	32	*aac(6′)-Ib, aac(6′)-Ib-cr, aadA2, aph(3″)-Ib, aph(6)-Id, arr-2, bla*_CTX-M-15_ *, bla*_TEM-1B_ *, dfrA12, mph(A), qnrS1, rmtB, sul1, sul2, tet(A)*	*bla*_NDM-5_	IncFIA, IncFIB, IncFII, IncY, Col (pHAD28)	IS*3*, IS*5075*, IS*Ec1*, IS*Ec9*, IS*Ec38*, IS*Ecl1*, IS*Kpn8*, IS*Kpn19*, IS*Sfl10*, MITE*Ec1,* IS*Aba125*	Tn*125*
					AGA0039.T1	1	*bla*_CTX-M-15_*, bla*_TEM-1B,_ *rmtB*	*bla*_NDM-5_	**IncFIA, IncFIB, IncFII (NDM)**	**IS*Aba125* (NDM)**	**Tn*125* (NDM)**
AGA0040	Urine	ST1588	D	O183:H34	Successful	16	*aadA2, bla*_CTX-M-15_ *, dfrA12, mph(A), qnrS1, sul1, tet(B)*	*bla*_NDM-5_	IncFIA, IncFII, IncX3, p0111	IS*3*, IS*26*, IS*100*, IS*421*, IS*609*, IS*6100*, IS*Ec1*, IS*Ec9*, IS*Kpn8*, MITE*Ec1,* IS*Aba125*	Tn*5403,* Tn*125*
					AGA0040.T1	8	*-*	*bla*_NDM-5_	**IncFIA (NDM)**	**IS*Aba125* (NDM)**	**Tn*125* (NDM)**
AGA0045	Urine	ST448	B1	O8:H8	Successful	32	*bla*_CMY-42_	*bla*_NDM-7_	IncX3	IS*4*, IS*5*, IS*26*, IS*102*, IS*911*, IS*Kpn8*, IS*Kox3*, IS*Ec17*, IS*Ec38*, MITE*Ec1*, IS*3000*	-
					AGA0045.T1	64	-	*bla*_NDM-7_	**IncX3 (NDM)**	**IS*3000* (NDM)**	-
AGA0047	Urine	ST68	D	O99:H6	Successful	32	*aadA2, aadA5, bla*_CTX-M-15_ *, bla_TEM-1B_, catA1, dfrA12, dfrA17, mph(A), rmtB, sul1*	*bla*_NDM-5_	IncFIA, IncFIB, IncFII, Col RNAI	IS*3*, IS*5*, IS*26*, IS*6100*, IS*Ec1*, IS*Ec18*, IS*Ec31*, IS*Ec38*, IS*Ec46*, MITE*Ec1,* ∆IS*Aba125*	∆Tn*125*
					AGA0047.T1	16	*bla*_TEM-1B_ *, rmtB*	*bla*_NDM-5_	**IncFIB, IncFII (NDM)**	**∆IS*Aba125* (NDM)**	**∆Tn*125* (NDM)**
AGP0009	Blood	ST68	D	O99:H6	Successful	8	*aadA2, aadA5, bla*_CTX-M-15,_ *bla*_TEM-1B_*, catA1, dfrA12, dfrA17, mph(A), rmtB, sul1*	*bla*_NDM-5_	IncFIA, IncFIB, IncFII, Col RNAI	IS*Ec46*, MITE*Ec1*, IS*5*, IS*Ec1*, IS*Ec31*, IS*Ec38*, IS*3*, IS*Ec18*, IS*6100,* ∆IS*Aba125*	∆Tn*125*
					AGP0009.T1	8	*bla*_CTX-M-15,_ *bla*_TEM-1B_	*bla*_NDM-5_	**IncFII (NDM)**	**∆IS*Aba125* (NDM)**	**∆Tn*125* (NDM)**
AGP0020	Blood	ST68	D	O99:H6	Successful	8	*aadA2, aadA5, bla*_CTX-M-15,_ *bla*_TEM-1B_*, catA1, dfrA12, dfrA17, mph(A), rmtB, sul1*	*bla*_NDM-5_	IncFIA, IncFIB, IncFII, ColRNAI	IS*3*, IS*5*, IS*26*, IS*6100*, IS*Ec1*, IS*Ec18*, IS*Ec31*, IS*Ec38*, IS*Ec46*, MITE*Ec1,* ∆IS*Aba125*	∆Tn*125*
					AGP0020.T1	64	*bla*_CTX-M-15,_ *bla*_TEM-1B_	*bla*_NDM-5_	**IncFIB, IncFII (NDM)**	**∆IS*Aba125* (NDM)**	**∆Tn*125* (NDM)**

MIC, Minimum inhibitory concentration; IS, Insertion sequence; WGS, Whole-genome sequencing; T, Transconjugants. IS elements, replicons, and transposons associated with carbapenemases have been bold faced.

Additionally, the isolates harbored different types of resistance genes such as: *bla*_CTX-M-15_, *bla*_OXA-1_, *bla*_TEM-1B,33,57_, *bla*_SHV-1,67,81,83,100,148,167_, *bla*_CMY-6,42,54_, *bla*_DHA-7_ (β-lactamase genes), *aac(6′)-Ib, aac(6′)-Ib-cr*, *rmtB*, *rmtC* (aminoglycoside resistance genes), *oqxA*, *oqxB*, *qnrS1*, and *aac(6′)-Ib-cr* (fluoroquinolone resistance genes). Apart from this, several other genes imparting resistance to different antimicrobials, such as chloramphenicol and trimethoprim, were found in the genomes (Tables 2, 3).

### 3.3 Molecular typing of carbapenemase-producing isolates

Carbapenemase-producing *K. pneumoniae* and *E. coli* were diverse as per PFGE band patterns (Figures 2B, 3B). However, in *E. coli*, two clonal clades were identified. In clade I, the clonal isolates (AGA0008, AGA0019, AGA0022, and AGA0028) were recovered from the same ICU (AICU), and the clones appear to persist in the ICU environment over 4 months. In clade II, two isolates (AGP0009 and AGA0018) were collected within 1 month from two different ICUs (PICU and AICU) while the other two isolates (AGA0047 and AGP0020) were collected after 3 and 7 months of the initial isolation, exhibiting the potential of this clone to spread and persist in both the ICUs (Supplementary Table S2). Such findings indicated an inter-ICU spread of carbapenemase-producing *E. coli* isolates (Table 3; Figure 3B).

Nine different STs, namely, ST16 (*n* = 2), ST101 (*n* = 2), ST231 (*n* = 2), ST2096 (*n* = 2), ST29, ST37, ST147, ST219, and ST437 (Table 2; Figure 2A), were noted in *K. pneumoniae* isolates, while eight different STs, namely, ST68 (*n* = 4), ST410 (*n* = 4), ST448 (*n* = 2), ST167, ST648, ST1588, ST2083, and ST2851 (Table 3; Figure 3A), were noted in *E. coli* isolates. A core phylogeny for *K. pneumoniae* and *E. coli* was carried out (Figures 2A, 3A). Branching patterns of the phylogeny trees for both *E. coli* and *K. pneumoniae* were similar to the PFGE cladogram for the majority of isolates, while they varied for a few (*E. coli*: AGA0014, AGA0040, and AGA0039; *K. pneumoniae*: AGA0024, AGA0042, AGA0058, AGA0077, and AGA0084). Core phylogeny of the *E. coli* isolates exhibited two clades, concordant with PFGE results (Figures 3A,B). These clades (clades I and II) were found to cluster ST410 (*n* = 4) and ST68 (*n* = 4), respectively, again reinforcing the fact of inter-ICU spread.

Apart from PFGE and MLST, K-/O-antigen (*K. pneumoniae*) and O-/H-antigen (*E. coli*) serotyping were performed. Study *K. pneumoniae* exhibited eight diverse serotypes. K64 is the prevalent serotype (*n* = 3) followed by K51 (*n* = 2) and K81 (*n* = 2) (Table 2). Among the *E. coli* isolates, O8 (*n* = 4) and O99 (*n* = 4) were the most commonly encountered serotypes (Table 3).

### 3.4 Transmissibility of carbapenemases

Conjugal transfer of carbapenemase genes (*bla*_NDM-1,5,7_) was successful in the majority of isolates. However, few isolates (AGA0008, AGA0019, and AGA0022) with *bla*_NDM-5_ did not show successful conjugation. Isolates co-harboring *bla*_NDM-5_ and *bla*_OXA-181_ transferred only *bla*_NDM-5_. None among *bla*_OXA-181,232_ and *bla*_KPC-2_ showed conjugal transfer (Tables 2, 3).

### 3.5 Genetic environment of carbapenemase genes and their association with different MGEs

Analysis of the genetic environment is based on findings from WGS data and is restricted to the size of the contigs containing the carbapenemase genes. Primer walking was performed as and when required. Transmissibility of the carbapenemase genes and association with different MGEs were assessed by conjugation and PCR-based assay.

#### 3.5.1 *bla*_NDM-1,5,7_

**Figure 4:**
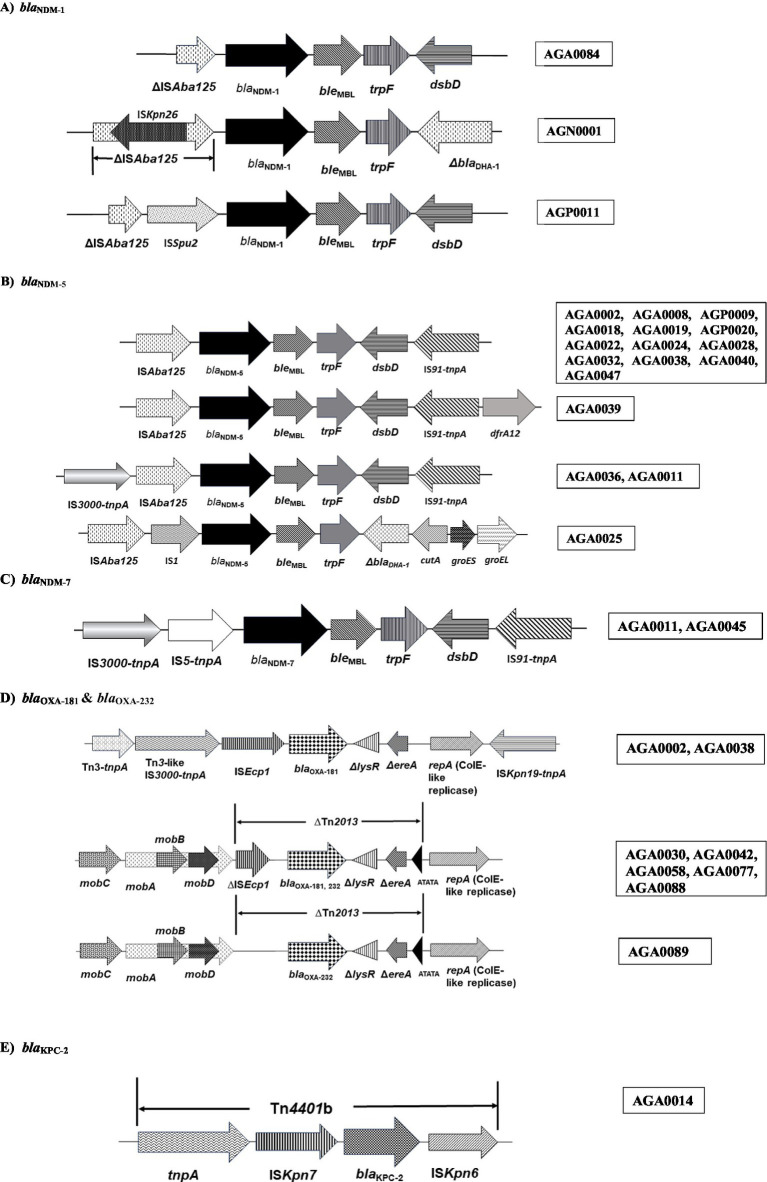
Schematic presentation of mobile genetic elements associated with carbapenemases found in this study. **(A)** *bla*_NDM-1_; **(B)** *bla*_NDM-5_; **(C)** *bla*_NDM-7_; **(D)** *bla*_OXA-181_ and *bla*_OXA-232_; **(E)** *bla*_KPC-2_. Target site duplications (ATATA) generated by the insertion of Tn*2013* are indicated by black triangles. *bla*, beta-lactamase; *mobA*, *mobB*, *mobC*, and *mobD*, mobilization relaxosome proteins; ∆*lysR*, truncated LysR-type transcriptional regulator; ∆*ereA*, truncated erythromycin esterase; *repA*, replicase; Tn, transposon; IS, insertion sequence; *tnpA*, transposase; *ble*_MBL_; bleomycin resistance gene; *trpF*, N-(5́-phosphoribosyl) anthranilate isomerase; *dsbD*, Disulfide bond oxidoreductase D; ∆, denotes deletion or truncation.

The genetic environment of *bla*_NDM-1_ and *bla*_NDM-5_ is similar in all the cases except for minute variations. The generic background noted in these isolates is in the following order: IS*Aba125*➔*bla*_NDM-1,5_➔bleomycin resssistance gene (ble_MBL_)➔phosphoribosyl anthranilate (*trpF*)➔disulfide reductase (*dsbD*) (Figures 4A,B). Truncation of IS*Aba125* with IS*Kpn26* (AGN0001), presence of IS*1* and IS*Spu2* between IS*Aba125* and *bla*_NDM-5_ have been noted in AGA0025 and AGP0011, respectively (Figures 4A,B). Downstream of *bla*_NDM-7_ exhibited similarity with *bla*_NDM-5_, but upstream of *bla*_NDM-7_ has IS*5*-like-*tnpA* followed by IS*3000*-*tnpA* instead of IS*Aba125* (Figure 4C). These differences in the IS elements have been depicted in Figures 4A–C.

*bla*_NDM_-bearing isolates harbor different types of plasmid replicons (Tables 2, 3; Figures 2A, 3A). The prevalence of IncFIA/FIB/FII and IncFIB has been noted in *E. coli* and *K. pneumoniae* isolates, respectively (Figures 2A, 3A). TCs harboring *bla*_NDM-1_ and *bla*_NDM-5_ exhibited the presence of IncFIB, IncFII, IncFIIK, IncN, IncR, and IncHI1B, IncN, IncX3, respectively, while IncX4 and IncI1γ were found in the TCs of *bla*_NDM-7_ (Tables 2, 3). Out of 12 *bla*_NDM-5_-possessing *E. coli*, 6 isolates exhibited a strong association of *bla*_NDM-5_ with IncFIA, IncFIB, and IncFII (Table 3).

Genomes of *bla*_NDM-1,5,7_ revealed the presence of different types of IS elements listed in Tables 2, 3. IS*1*, IS*Kpn26*, IS*Aba125*, IS*Spu2*, and IS*3000* were found in the immediate upstream of the *bla*_NDM_ and were associated with *bla*_NDM_ (Figures 4A–C).

Other than IS elements, NDM-harboring isolates carried different types of transposons, not necessarily associated with *bla*_NDM_ (Tables 2, 3). Of these transposons, Tn*125*, Tn*5403*, and Tn*6196* were found in both the study isolates, *E. coli* and *K. pneumoniae*. However, Tn*125* was found to be associated with NDM among the study isolates (Tables 2, 3).

#### 3.5.2 *bla*_OXA-181, 232_

*bla*_OXA-181,232_, found in this study, exhibited a similar genetic background. The presence of a mobilization relaxosome, followed by an intact/ truncated IS*Ecp1*, was noted upstream, and a truncated *lysR*, truncated *ereA*, and a replicase protein (ColKP3) downstream of *bla*_OXA-181,232_, except for one isolate (AGA0089) with deletion of IS*Ecp1* (Table 2; Figure 4D).

Similar to *bla*_NDM_-producing isolates, *bla*_OXA-181,232_-producing isolates also harbor different types of plasmid replicons (Table 2), predominantly IncFIB and ColKP3 (Figure 2A). Conjugal transfer of *bla*_OXA-181,232_ was unsuccessful, and the presence of ColKP3 was noted downstream of *bla*_OXA-181,232_ as per WGS data and primer walking.

*bla*_OXA-181,232_-harboring isolates exhibited the presence of different types of IS elements and transposons (Table 2). ColKP3, IS*Ecp1*, and Tn*2013* were associated with *bla*_OXA-181,232_ within the study isolates.

#### 3.5.3 *bla*_KPC-2_

One *bla*_KPC-2_-bearing *E. coli* had *bla*_KPC-2_ bracketed between IS*Kpn7* (upstream) and IS*Kpn6* (downstream) of Tn*4401*b (Figure 4E). This isolate harbored various IS elements and replicon types (Table 3). However, conjugal transfer of *bla*_KPC-2_ was unsuccessful; hence, the replicon associated with *bla*_KPC-2_ in the study isolate could not be determined.

### 3.6 Comparative genome analysis of study isolates with relevant carbapenemase-producing isolates

**Figure 5:**
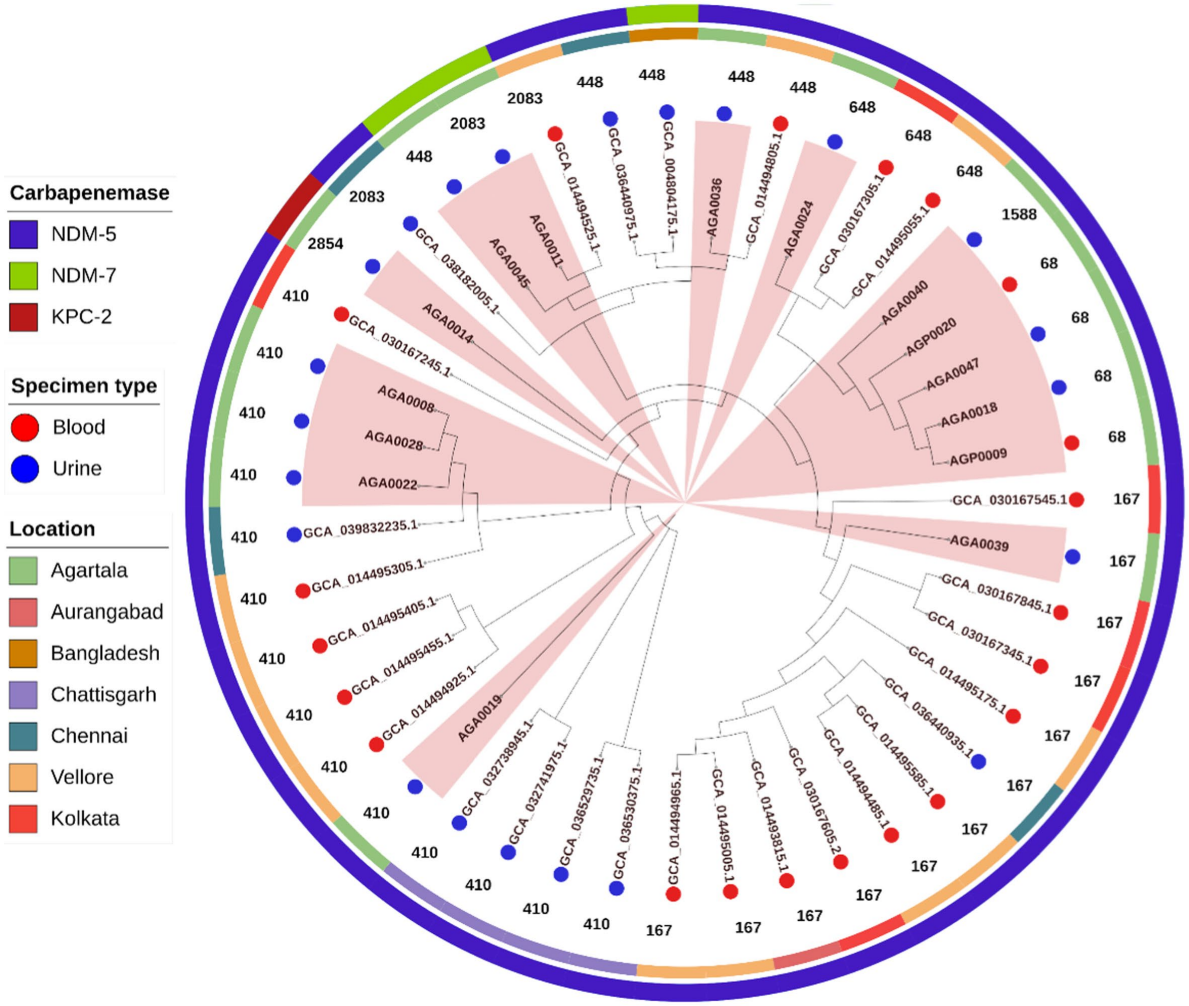
Core genome phylogeny of 43 *E. coli* using Roary (v3.13.0) and iTOL (v7). Isolates are colored at the endpoint according to specimen type, followed by sequence types (in numerical value). The additional two outer circles denote the place of collection (inner circle) and the presence of *bla*_NDM_ variants and *bla*_KPC-2_ carbapenemases (outer circle). Clades containing isolates from this study are highlighted in peach.

**Figure 6:**
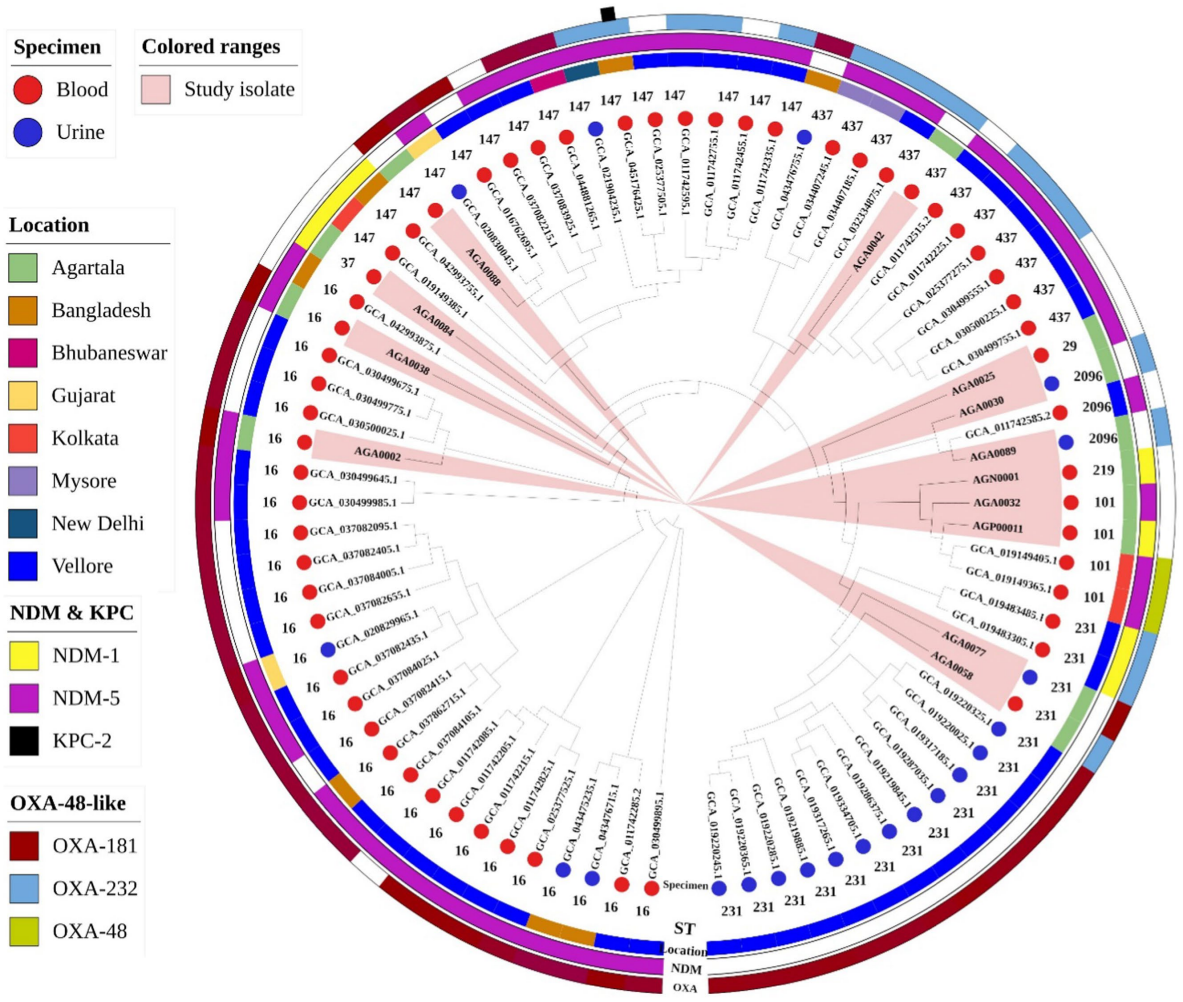
Core genome phylogeny of 79 *Klebsiella pneumoniae* using Roary (v3.13.0) and iTOL (v7). Isolates are colored at the endpoint according to specimen type, followed by sequence types (in numerical value). The additional three outer rings (from inside to the out): circle-1 represents the location of collection (inner circle), circle-2 represents class-B carbapenemases (middle circle), and circle 3 represents class-D carbapenemases (outer circle). KPC-2 was present in one isolate and has been described as a black box outside the outer ring. Clades containing isolates from this study are highlighted in peach.

Core genome phylogeny trees for *E. coli* (Figure 5) and *K. pneumoniae* (Figure 6) were prepared with isolates from different parts of India and Bangladesh. Both *E. coli* and *K. pneumoniae* study isolates showed relatedness with other isolates from Chhattisgarh, New Delhi, Gujarat, Chennai, Kolkata, Vellore, Mysore, and Bhubaneswar, and the neighboring country, Bangladesh. The phylogenetic tree for *E. coli* comprised 43 isolates, including 15 isolates from this study (Figure 5). Study isolates were diverse and scattered throughout the phylogeny. Isolates for this analysis were equally distributed between blood (*n* = 21) and urine (*n* = 22). In this collection, the prevalence of two epidemic clones, such as ST410 (*n* = 14, isolated from blood and urine) and ST167 (*n* = 12, from blood), was noted. It was noted that all isolates, that is, both study and others, primarily harbored *bla*_NDM-5_ (*n* = 39) with few *bla*_NDM-7_ (*n* = 3), and *bla*_KPC-2_ was only found in the study isolate, AGA0014. One uropathogenic *E. coli* (AGA0036) from this study was distantly related to an uropathogenic isolate from Bangladesh. Mostly, the study isolates from blood and urine showed relatedness with other Indian isolates of blood and urine origin, respectively. Although exceptions were noted, wherein uropathogenic study isolates (AGA0011, AGA0024, AGA0036, and AGA0039) showed relatedness with other Indian isolates of blood origin.

The phylogenetic tree of *K. pneumoniae* comprised 79 isolates, including 13 isolates from this study (Figure 6). Similar to the *E. coli* study isolates, *K. pneumoniae* was also diverse. Unlike the phylogenetic tree for *E. coli* (Figure 5), the *K. pneumoniae* phylogenetic tree was primarily composed of isolates from blood (*n* = 58/79, 73%). AMR high-risk clones ST16 (*n* = 27) is the dominant ST, followed by ST231 (*n* = 16) and ST147 (*n* = 15) distributed between both blood and urine. In this collection, two carbapenemases, namely, *bla*_NDM-5_ (*n* = 43) and *bla*_OXA-181_ (*n* = 44) are the prevalent ones followed by isolates harboring multiple carbapenemases, namely, *bla*_NDM-1_ and *bla*_OXA-232_ (*n* = 2), *bla*_NDM-5_ and *bla*_OXA-181_ (*n* = 20), *bla*_NDM-5_ and *bla*_OXA-48_ (*n* = 2). The presence of *bla*_NDM-5_ along with *bla*_KPC-2_ (*n* = 1) was rarely noted. Multiple carbapenemase-harboring isolates were largely of blood origin. Analogous to the phylogenetic tree of *E. coli*, relatedness of study isolates from urine (AGA0058 and AGA0089) and blood (AGA0088) with isolates from India (blood) and Bangladesh (urine), respectively, was observed (Figure 6).

## 4 Discussion

HGT, an essential driver of bacterial evolution along with its mediators, MGEs, has mobilized different genes, which have benefited other bacteria to meet the evolutionary challenges of combating antimicrobials (Soucy et al., 2015). A web of genomic exchange has been noted among organisms, which has not been limited to species but has traversed to different genera and even phyla, indicating a re-evaluation of the “species concept” (Goldenfeld and Woese, 2007). Involvement of MGEs in the spread of carbapenem resistance has been documented worldwide, including various regions of India. As MGEs can facilitate the spread of resistance determinants between isolates and even species, studying them is crucial for understanding the transmission of resistance. Hence, this study assessed carbapenemase-associated MGEs and their interplay in the spread of carbapenem resistance within *E. coli* and *K. pneumoniae*.

Study isolates exhibited moderate (40%) to high (68%) level resistance to carbapenems, which is in contrast with previous reports from Northeast India, wherein low-level resistance (5–19%) to carbapenems was noted among Enterobacterales (Chellapandi et al., 2017; Sharma et al., 2021; Ralte et al., 2022). Study isolates carried many resistance genes, emphasizing the MDR nature of the isolates. Three carbapenemases (*bla*_NDM-1,5,7_, *bla*_KPC-2_, and *bla*_OXA-181,232_) were noted in this study. The prevalence of *bla*_NDM-5_ in both genera of this study indicated an allelic shift of *bla*_NDM-1_ to *bla*_NDM-5_, a phenomenon also documented in other global studies (Wu et al., 2019). NDM-5 has two mutations, V88L and M154L, compared to NDM-1 (Wu et al., 2019). V88L has been reported to exhibit 4-fold higher carbapenemase activity than NDM-1 (Hornsey et al., 2011). Further, M154L is responsible for higher zinc affinity, providing stability to the enzyme, thereby conferring higher resistance than NDM-1 (Bahr et al., 2018). Enhanced stability and increased carbapenemase activity of *bla*_NDM-5_ perhaps established this as a dominant allele over *bla*_NDM-1_. Report of *bla*_KPC-2_-harboring *E. coli* has been documented from India (Das et al., 2021) with no available genome description in public repositories such as NCBI. To our knowledge, this is the first study from India documenting a *bla*_KPC-2_-harboring *E. coli* genome.

In this study, isolates co-harboring *bla*_NDM-5_ and *bla*_OXA-181_ exhibited increased meropenem MIC (>128 mg/L), as also noted in previous studies (Naha et al., 2021; Bianco et al., 2022). The variability of carbapenemases was higher in isolates from AICU, as overall isolation of Enterobacterales was higher in this ICU than others. Previous studies from this region documented the presence of a single carbapenemase, that is, *bla*_NDM-1_ (Mukherjee et al., 2019) or *bla*_OXA-48_ (Ralte et al., 2022) or *bla*_KPC-2_ (Das et al., 2021). However, this study reports the presence of multiple carbapenemases (*bla*_NDM-1,5,7_, *bla*_KPC-2_, and *bla*_OXA-181,232_) from a single hospital and also the presence of dual carbapenemases within an isolate. The presence of multiple carbapenemases warrants a threat, and strict infection control needs to be ensured.

Study isolates were diverse, as found from PFGE and core genome phylogeny, and belonged to different STs. Differences in branching patterns for core phylogeny and PFGE noted in very few isolates of this study are due to the fact that PFGE and core genome phylogeny use two different approaches for genomic typing. PFGE compares band patterns generated due to the enzymatic digestion of genomic DNA, leading to a less significant view of genomic differences between isolates. On the other hand, core genome phylogeny is based on the sequences of core genes present in the isolates, providing a more detailed depiction of genetic relatedness (Gona et al., 2020). In this study, carbapenemases were found in high-risk international clones of *K. pneumoniae* (ST16, ST101, ST147, and ST231) and uropathogenic high-risk MDR clones of *E. coli* (ST167, ST410, and ST648), as also reported in other studies (Nittayasut et al., 2021; Bhattacharjee et al., 2023) highlighting their propensity for transboundary spread. *K. pneumoniae* ST2096 isolated from the ICUs is now being considered as an emerging hypervirulent MDR clone worldwide (Shankar et al., 2022). The presence of hypervirulent serotypes, such as K2 and K64, in high-risk international clones, ST101 and ST147, respectively, increases the spread of resistance and virulence. Association of different carbapenemases with the STs, as seen in this study, corroborates findings from various parts of India and the globe (Naha et al., 2021, 2022; Bhattacharjee et al., 2023; Shukla et al., 2023). The diversity of study isolates signifies that the spread of carbapenemases is not through specific clones or lineages, but via involvement of MGEs.

Comparative core genome phylogenetic analysis showed similarities of study isolates with isolates from the Indian cities - Kolkata, Chennai, Vellore, Aurangabad, Mysore, Bhubaneshwar, and the state, Chhattisgarh along with the neighboring country, Bangladesh. Similarities of isolates from blood with those from urine, as seen through the phylogenetic analysis, indicated the potential of the study isolates to cause invasive systemic infections (Shah et al., 2022; Bhattacharjee et al., 2023; Mueller and Tainter, 2025). India is a large country with distinct geographies and also sharp differences in health indicators. In spite of such differences, isolates exhibited relatedness with other Indian isolates, which is indicative of the spread of these isolates across different parts of India.

Study genomes harbored an assemblage of different plasmids. Study *E. coli* exhibited dominant association of *bla*_NDM_ with IncFIA, IncFIB, IncFII (primarily), along with IncX3, IncX4, IncI1γ. On the other hand, study *K. pneumoniae* showed an association of *bla*_NDM_ with different replicons such as IncFIA, IncFIB, IncFII, IncN, IncR, and IncHI1B. Globally, *bla*_NDM_ has been reported in at least 20 types of replicons (Wu et al., 2019) with continent-specific dominance of certain replicons, namely, America (IncFI), Asia (South, East, and West) (IncA/C, IncR, IncHI2, IncX3, and IncY), and Europe (IncX1/X4/X6) (Li et al., 2023). Previous studies have noted association of NDM with F-plasmids (Partridge et al., 2018) along with IncX3 and IncHI1 replicons (Wu et al., 2019). Recent studies have documented association of NDM with the hybrid IncFIA/FIB/FII plasmid (Zhao et al., 2022; Bhattacharjee et al., 2023), as also noted in isolates from this study. Conjugal transfer and involvement of NDM with diverse replicons among the study isolates highlighted the dissemination potential of *bla*_NDM_ across various genera.

Recently, it has been noted that *bla*_NDM_-bearing plasmids harbor different virulence genes along with ARGs, forming a sizeable hybrid plasmid (>200 kb). pNDM-MAR is one such plasmid (Villa et al., 2012), which is noted in one study isolate (AGA0089), composed of two replicons, namely, IncFIB and IncHI1B. Virulence and resistance in the same plasmid allow bacteria to persist, colonize, and spread resistance in hostile conditions. In contrast to *bla*_NDM_, *bla*_OXA-181,232_ found in the study isolates is restricted to the non-conjugative ColKP3 replicon, corroborating with previous reports (Potron et al., 2011; Pitout et al., 2019; Shankar et al., 2019; Naha et al., 2021). *bla*_OXA-181,232_ has been reported in limited numbers of plasmid replicons (ColE2, IncN1, IncT, IncX3, and ColKP3), majority of which are non-self-transmissible. Previous studies have shown transfer of *bla*_OXA-181,232_ plasmids with aid of helper plasmids (Pitout et al., 2019; Naha et al., 2021). In addition, clonal outbreaks of *bla*_OXA-181,232_ have also been reported (Pitout et al., 2019; Guzmán-Puche et al., 2021; Boyd et al., 2022). The study isolates co-harboring *bla*_OXA-181_ and *bla*_NDM-5_, did not show transfer of *bla*_OXA-181_. *bla*_OXA-181,232_ and *bla*_NDM-5_ were present on separate plasmids, suggesting an independent acquisition of two carbapenemase-harboring plasmids at different time points. Restricted mobility of the Col plasmids probably limited the spread of this gene compared to *bla*_NDM_. Another carbapenemase, *bla*_KPC-2_, was found in one *E. coli*, borne on a non-conjugative plasmid, as documented in a previous study (Naha et al., 2020). WGS data revealed the presence of various replicons in this isolate, but the replicon associated with *bla*_KPC-2_ could not be determined due to the non-conjugative nature of the plasmid and the limitation of short-read sequencing.

Association of IS elements and transposons with the carbapenemases has been noted in this study. Carbapenemases (*bla*_NDM_, *bla*_OXA-181,232_, and *bla*_KPC-2_) exhibited an ancestral genetic context. *bla*_NDM_ was bracketed between IS*Aba125* and *ble*_MBL_, corroborating with other global studies, implying a prominent role of the IS element in the mobilization of this gene (Acman et al., 2022). IS*Aba125*, being part of Tn*125*, indicates the active role of this transposon in the spread of *bla*_NDM_, as also documented in previous studies (Wu et al., 2019; Acman et al., 2022; Li et al., 2023). Tn*125* has undergone minute variations leading to a complex genetic context (truncation by two copies of the same IS elements, such as IS*26*, IS*903*, and IS*3000*) (Toleman et al., 2012), but over the years the mobility of this transposon has been retained.

*bla*_OXA-181,232_ were present in association with an intact or truncated IS*Ecp1* and ∆*lysR-*∆*ereA* upstream and downstream, respectively, on an intact or truncated Tn*2013* of a ColKP3 plasmid, comparable to studies from various parts of the world (Pitout et al., 2019; Naha et al., 2021). Deletion/insertion and truncation of this IS element restrict the spread of these genes (Pitout et al., 2019; Naha et al., 2021). In contrast to the results of this study and other global data, a study on MGEs of *bla*_OXA-181,232_ from North and South India exhibited a highly diverse genetic background involving different IS elements such as IS*X4*, IS*1*, IS*3*, IS*Kpn*1, etc. (Shankar et al., 2019). It has been found that the spread of *bla*_OXA-181,232_ among Enterobacterales is driven by IS*Ecp1* (Pitout et al., 2019). *bla*_OXA-181,232_ has been associated with different types of transposons, namely, Tn*2013*, Tn*2016*, Tn*6360,* Tn*6237*, and Tn*51098* (Pitout et al., 2019) but the occurrence of IS*Ecp1* among the study isolates indicated the presence of *bla*_OXA-181,232_ within Tn*2013*, the widely reported transposon responsible for both transboundary and interspecies spread of *bla*_OXA-181,232_ among Enterobacterales (Pitout et al., 2019). *bla*_KPC-2_ in this study has been found between IS*Kpn7* and IS*Kpn6* within Tn*4401*b. Out of 8 variants of Tn*4401* (Tn*4401*a- Tn*4401*h) (Tang et al., 2022), Tn*4401*b has been considered to play a crucial role in the spread of *bla*_KPC-2_, while other transposons such as Tn*1721*, and IS*26*-like transposon are also emerging as vehicles for dissemination of *bla*_KPC-2_ in Southern Asia (Tang et al., 2022). The study isolate retained involvement of Tn*4401*b, similar to a previous study from India (Naha et al., 2020).

This study reports various MGEs, which included both plasmids and transposons associated with carbapenem-resistant genes. The spread of *bla*_NDM_ across several countries within 15 years is evidence of the capability of the MGEs (such as Tn*3*, IS*5*, IS*26*, IS*Aba125*, IncF-type, and IncX-type plasmids) in the spread of this gene. Initial studies indicated that the IS*Aba125* is solely responsible for the spread of *bla*_NDM_, but lately other IS elements such as IS*26* have gained attention (Wu et al., 2019). IS*26* is one such MGE readily found in the majority of the genomes. IS*26* is responsible for the formation of large fusion plasmids harboring a collection of resistance genes, sometimes also generating tandem repeats of resistance genes such as *bla*_NDM_ in a single plasmid. It can also integrate into the promoter region of a gene and can cause constitutive expression of the gene, leading to increased resistance (Harmer and Hall, 2024). Association of *bla*_NDM_ with specific plasmids (primarily IncF plasmids) has also played a significant role in its spread. Contrary to *bla*_NDM_, the spread of *bla*_OXA-181,232_ and *bla*_KPC-2_ has been restricted to specific geographical regions with few sporadic cases in other parts of the globe (Pitout et al., 2019; Ding et al., 2023). This study also noted that the spread of *bla*_NDM_ has primarily been driven by plasmids and IS elements, but in the case of *bla*_OXA-181,232_ and *bla*_KPC-2_, though MGEs play an essential role, the spread of these carbapenemases is associated with specific clonal lineages. Spread of KPC through sequence type 258 (ST258) and ST11 has been reported worldwide (Marsh et al., 2019; Yuan et al., 2024). Global dissemination of OXA-48 and its derivatives is often associated with specific high-risk clones, such as ST14, ST15, ST147, ST231, ST307 (*K. pneumoniae*), and ST38 and ST410 (*E. coli*) (Pitout et al., 2019). The contribution of MGEs is more critical in the spread of resistance than bacterial lineages, leading to the emergence of new resistant clones (Caliskan-Aydogan and Alocilja, 2023).

In conclusion, this study highlighted the association of HGT and different MGEs in the mobilization of carbapenemases. The use of short-read sequencing limits the detailed characterization of the carbapenem-resistant plasmids. Similarity of MGEs (plasmid profiles, transposons, and IS elements) for the carbapenemases (particularly *bla*_NDM_) of this study is concordant with reports across the globe indicating their efficiency to transfer in diverse lineages and existence as part of the global gene pool. Other than this, MGEs also provide information about the local selective pressures that influence gene distribution via HGT. The occurrence of fusion plasmids (pNDM-MAR) exhibiting integration of virulence with resistance genes has already been noted. The presence of such plasmids is worrisome as this expedite the spread of carbapenem resistance and virulence. On this account, surveillance of these genes and MGEs remains very relevant for infection control.

## Data Availability

The datasets presented in this study can be found in online repositories. Genome sequence data have been deposited at NCBI under BioProject No. PRJNA790720. Antimicrobial resistance pattern of *E. coli & K. pneumoniae* and detailed patient data can be found in the Supplementary material.

## Footnotes

^1^ https://iris.who.int/bitstream/handle/10665/376776/9789240093461-eng.pdf

^2^ https://static.pib.gov.in/WriteReadData/specificdocs/documents/2023/jun/doc202362208601.pdf

^3^ https://www.diatheva.com/product/pbrt-2-0-kit/

^4^ https://usegalaxy.eu/

^5^ https://rast.nmpdr.org/

^6^ https://card.mcmaster.ca/

^7^ https://pathogen.watch/

^8^ https://cge.food.dtu.dk/services/PlasmidFinder/

^9^ https://cge.food.dtu.dk/services/SerotypeFinder/

^10^ https://cge.food.dtu.dk/services/MobileElementFinder/
