# Supplementary material for: Diversity of mobile genetic elements in carbapenem-resistant Enterobacterales isolated from the intensive care units of a tertiary care hospital in Northeast India
Source: Front Microbiol. 2025 May 22;16:1543427. doi: 10.3389/fmicb.2025.1543427 (PMC12137328; doi:10.3389/fmicb.2025.1543427)
Supplement: Supplementary file 1 [file Table_1.docx]

**Table S1: Comparison of antimicrobial resistance pattern in *Escherichia coli* and *Klebsiella pneumoniae* isolated from various intensive care units**

| Antibiotics | *E. coli* (n=48) | *K. pneumoniae* (n=22) |
| --- | --- | --- |
| TIC/CLA | 69% | 77% |
| PIP/TA | 56% | 73% |
| CTZ | 81% | 73% |
| CEF/SUL | 52% | 68% |
| CFM | 58% | 68% |
| AZT | 81% | 82% |
| DOR | 40% | 68% |
| IMP | 40% | 68% |
| MER | 40% | 68% |
| AMK | 17% | 55% |
| GEN | 40% | 46% |
| CIP | 90% | 77% |
| LEV | 94% | 77% |
| MNC | 50% | 68% |
| COL | 2% | 14% |
| SXT | 63% | 41% |

Abbreviation: Ticarcillin/clavulanic acid (TIC/CLA), piperacillin/tazobactam (PIP/TA), ceftazidime (CTZ), cefoperazone/sulbactam (CEF/SUL), cefepime (CFM), aztreonam (AZT), doripenem (DOR), imipenem (IMP), meropenem (MER), amikacin (AMK), gentamicin (GEN), ciprofloxacin (CIP), levofloxacin (LEV), minocycline (MNC), colistin (COL), trimethoprim/sulfamethoxazole (SXT).

**Table S2: Demographic data and clinical features of the patient from whom *Klebsiella pneumoniae* and *Escherichia coli* have been collected.**

| **Isolate identified** | **Patient Id** | **Date of admission** | **ICU type** | **Specimen type** | **Gender** | **Age** | **Diagnosis at admission** | **Antibiotics used** | **Final outcome** |
| --- | --- | --- | --- | --- | --- | --- | --- | --- | --- |
| ***Klebsiella pneumoniae*** | AGA0002 | October, 2019 | Anaesthesia | Blood | Female | 68 yrs | Organ Failure | Piperacillin-tazobactam | Not known |
|  | AGA0030 | October, 2020 | Anaesthesia | Blood | Male | 69 yrs | Chronic kidney disease with Diabetes mellitus type-2, Cerebrovascular accident | Amikacin, cefotaxime | Discharged |
|  | AGA0032 | October, 2019 | Anaesthesia | Blood | Male | 57 yrs | Diabetes mellitus type-2 with Acute kidney disease | Amikacin | Discharged |
|  | AGA0038 | March, 2020 | Anaesthesia | Blood | Female | 42 yrs | Stroke | Imipenem | Discharged |
|  | AGA0042 | February, 2020 | Anaesthesia | Blood | Female | 81 yrs | Seizure, Acute Infarction | Meropenem | Discharged |
|  | AGA0077 | January, 2021 | Anaesthesia | Blood | Male | 65 yrs | Chronic kidney disease with Diabetes mellitus type-2 | Piperacillin-tazobactam | Discharged |
|  | AGA0084 | March, 2021 | Anaesthesia | Blood | Male | 55 yrs | Not mentioned | Piperacillin-tazobactam, meropenem | Discharged |
|  | AGA0088 | March, 2021 | Anaesthesia | Blood | Male | 62 yrs | Involuntary jerky movement | Meropenem | Discharged |
|  | AGN0001 | November, 2019 | Neonatal | Blood | Male | 1 Day | Respiratory distress | Amikacin, cefotaxime | Discharged |
|  | AGP0011 | March, 2020 | Paediatric | Blood | Female | 2 Days | Perinatal asphyxia, respiratory distress | Amikacin, cefotaxime | Expired |
|  | AGA0025 | October, 2020 | Anaesthesia | Urine | Female | 29 yrs | Breathlessness | Amikacin, cefotaxime | Discharged |
|  | AGA0058 | July, 2020 | Anaesthesia | Urine | Male | 54 yrs | Chronic kidney disease with Diabetes mellitus type-2 | Meropenem | Not known |
|  | AGA0089 | April, 2021 | Anaesthesia | Urine | Female | 48 yrs | Myocardial infarction | Linezolid, doxycycline | Discharged |
| ***Escherichia coli*** | AGA0008 | October, 2019 | Anaesthesia | Urine | Female | 40 yrs | Fever, malaise | Ceftriaxone | Discharged |
|  | AGA0011 | December, 2019 | Anaesthesia | Urine | Female | 20 yrs | Psychogalvanic reaction with anaemia | Artesunate (malarial drug) | Not known |
|  | AGA0014 | December,2019 | Anaesthesia | Urine | Female | 51 yrs | Fever with abdominal pain | Ceftriaxone | Discharged |
|  | AGA0018 | January, 2020 | Anaesthesia | Urine | Female | 20 yrs | Fever, pelvic pain | Amikacin, cefotaxime | Discharged |
|  | AGA0019 | January, 2020 | Anaesthesia | Urine | Male | 63 yrs | Right-sided hydropneumothorax | Piperacillin-tazobactam, meropenem, moxifloxacin, cindamycin | Discharged |
|  | AGA0022 | January, 2020 | Anaesthesia | Urine | Female | 27 yrs | Shortness of breath, Fever | Nitrofurantoin, Meropenem | Discharged |
|  | AGA0024 | January, 2020 | Anaesthesia | Urine | Female | 57 yrs | Shortness of breath, Fever | Meropenem, piperacillin tazobactam | Discharged |
|  | AGA0028 | February, 2020 | Anaesthesia | Urine | Female | 48 yrs | Tuberculosis, meningitis | Nitrofurantoin, Meropenem | Discharged |
|  | AGA0036 | November, 2019 | Anaesthesia | Urine | Male | 65 yrs | Abdominal pain | Meropenem | Discharged |
|  | AGA0039 | February, 2020 | Anaesthesia | Urine | Male | 58 yrs | Slurring of speech | Piperacillin tazobactam | Discharged |
|  | AGA0040 | March, 2020 | Anaesthesia | Urine | Female | 77 yrs | Heart failure | Cefoperazone | Discharged |
|  | AGA0045 | March, 2020 | Anaesthesia | Urine | Male | 48 yrs | Abdominal Pain | Cefoperazone | Expired |
|  | AGA0047 | March, 2020 | Anaesthesia | Urine | Male | 22 yrs | Breathlessness | Linezolid, doxycycline | Expired |
|  | AGP0009 | December, 2019 | Paediatric | Blood | Female | 3 Months | Bloodstream infection | Not mentioned | Not known |
|  | AGP0020 | July, 2020 | Paediatric | Blood | Male | 1 yr | Distension of abdomen | Cefotaxime | Discharged |

Abbreviations: Intensive care unit (ICU), years (yrs)
